# Clinical Features and Outcomes of Patients with Idiopathic Inflammatory Myositis-Associated Interstitial Lung Disease in Rural Appalachia: A Cross-Sectional Study

**DOI:** 10.3390/jcm13051294

**Published:** 2024-02-25

**Authors:** Vishal Deepak, Bhanusowmya Buragamadagu, Fnu Rida Ul Jannat, Rachel Salyer, Ty Landis, Sayanika Kaur, Bathmapriya Balakrishnan

**Affiliations:** 1 Division of Pulmonary, Critical Care and Sleep Medicine, Department of Medicine, School of Medicine, West Virginia University, Morgantown, WV 26506, USA; 2 Division of Pulmonary, Critical Care and Sleep Medicine, Department of Medicine, New York University School of Medicine, New York, NY 10017, USA; 3 Department of Medicine, School of Medicine, West Virginia University, Morgantown, WV 26506, USA; 4 Division of Rheumatology, Department of Medicine, Indiana University School of Medicine, Indianapolis, IN 46202, USA; 5 Respiratory Institute, Cleveland Clinic, Cleveland, OH 44195, USA; balakrb@ccf.org

**Keywords:** idiopathic inflammatory myositis, interstitial lung disease, rural, mortality, hypoalbuminemia

## Abstract

Background: Idiopathic inflammatory myopathies (IIMs) are a group of autoimmune disorders often complicated by interstitial lung disease (ILD). The clinical characteristics and outcomes of IIM-associated ILD have been reported variably, but the literature on rural populations is scarce. Methods: A retrospective cross-sectional study was conducted at a rural tertiary academic medical center. Twenty-nine patients met the final inclusion criteria. The primary outcome was to assess the disease state and immunological and radiographic features of IIM-associated ILD. Secondary outcomes included disease progression, ILD exacerbation, mortality rate, and factors associated with poor outcome. Results: Dermatomyositis (n = 15, 51.72%) followed by polymyositis (n = 8, 27.58%) were predominant myopathies. The most common autoantibodies were anti-Jo1 antibodies (n = 11, 37.93%). Indeterminate usual interstitial pneumonitis (41.30%, n = 12) was the most common radiographic pattern followed by non-specific interstitial pneumonia (n = 5, 17.24%). ILD exacerbation (n = 14, 66.66%) and mortality rate (n = 6, 20.69%) were high. Albumin levels were significantly lower in patients who died. Conclusions: The clinical characteristics of patients with IIM-associated ILD in rural Appalachia exhibit notable distinctions, and outcomes are worse compared to other populations. Larger studies are needed to investigate other prognostics factors and longitudinal trends of clinical characteristics and outcomes of IIM-associated ILD in rural populations.

## 1. Introduction

Idiopathic inflammatory myopathies (IIMs) are a spectrum of systemic autoimmune disorders. Subtypes of IIMs include dermatomyositis (DM), polymyositis (PM), anti-synthetase syndrome (AS), inclusion body myositis, immune-mediated necrotizing myositis, and amyopathic dermatomyositis (ADM) [1]. The annual prevalence of IIM is estimated to range from 2.4 to 33.8 per 100,000 population and its incidence is reported to be 1.16–19 per million/year [2]. IIM is characterized by inflammation of the proximal muscles, elevated muscle enzyme levels, and weakness. Skin rash, interstitial lung disease (ILD), joint pains, vasculitis, dysphagia, and conduction abnormalities in the heart are some of the reported extra-muscular manifestations of IIM [3]. The mortality rate in IIM has been reported in different populations and may be as high as 26% [4,5]. Several factors including malignancy and cardiac and pulmonary complications have been key drivers of increased mortality rates in IIM [6].

ILD is a commonly recognized complication of myositis. It was first described in 1956 [7]. The estimated prevalence of IIM-associated ILD is variable and ranges between 5 and 78% [8,9,10,11] depending on the population studied and method of ascertainment. Clinical features range from asymptomatic to severe fulminant respiratory failure, similar to acute respiratory distress syndrome [12]. ILD in myositis has been reported to be the primary cause of death and hospitalizations due to respiratory failure [13]. Furthermore, lung disease may be an isolated manifestation of underlying IIM with subtle or late-onset extrapulmonary symptoms, resulting in delayed and/or misdiagnosis and inadequate treatment [14,15]. In patients with IIM, those with ILD have worse prognosis and significantly higher mortality in contrast to those without ILD [13,16,17].

Several studies have been conducted in different regions of the world looking at the clinical characteristics of patients with IIM, revealing contrasting results for IIM in general and IIM associated with ILD [18,19,20,21,22]. The differences in the prevalence, clinical characteristics, and outcomes in earlier studies underscore the complexity of this entity due to the variability of geographic, socioeconomic, and ethnic factors in these patients. The rural Appalachian region, due to unique environmental and socioeconomic factors and health care disparities, has an increased burden of pulmonary diseases [23]. Thus, it is foreseeable that the clinical characteristics and outcomes of IIM-ILD in this region may differ. To address the dearth of data in this population, we sought to determine the clinic–radiological characteristics, treatment response, and outcomes of the IIM-ILD rural Appalachian cohort. The identification of these features will add granularity of the distinctive features of patients with IIM-ILD in this cohort. In particular, we sought to determine specific extrapulmonary features that may lead to poor prognostic extrapulmonary features in patients with IIM-ILD. We aim to determine unique patterns of the presentation and course of patients with IIM-ILD.

## 2. Materials and Methods

### 2.1. Study Design and Setting

This was a retrospective cross-sectional study at a rural Appalachian tertiary academic center and its affiliated hospitals to observe the outcomes of patients with IIM-ILD. The study protocol was reviewed and approved by the West Virginia University (WVU) Institutional Review Board (IRB) (#2210659486) on 9 December 2022. The study was conducted in accordance with the ethical standards of IRB on human experimentation with the Helsinki Declaration of 1975. The study was reported in line with the Strengthening the Reporting of Observational Studies in Epidemiology (STROBE) guidelines [24].

### 2.2. Participants and Study Size

**Figure 1:**
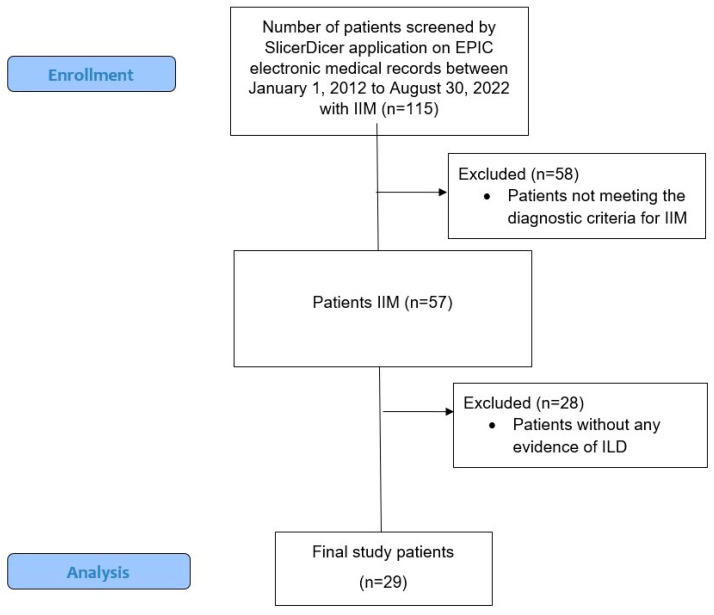
Consort flow diagram. IIM, idiopathic inflammatory myositis; ILD, interstitial lung disease.

The study utilized Slicer Dicer application for screening patients with the diagnosis of any IIM between 1 January 2012 to 30 August 2022. The Slicer Dicer is an inbuilt data exploration, analytic and reporting software of the EPIC electronic health record. It helps healthcare professionals and researchers to filter patient data on the basis of clinical endpoints such as diagnosis, treatments, procedures, etc., and analyze them effectively. The selection process of the patients is illustrated in a consort flow diagram (Figure 1) [25]. The study was conducted in line with “Sex and Gender Equity in Research” (SAGER) guidelines [26]. Patient genders were self-reported. No participants were excluded based on their gender. Participants who did not meet the European League Against Rheumatism (EULAR)/American College of Rheumatology (ACR) classification criteria for IIM were excluded. Additionally, patients with IIM but without evidence of ILD were excluded. Specifically, 86 patients were excluded due to lack of the following criteria: (1) definitive diagnosis of IIM based on EULAR/ACR criteria, (2) evidence of ILD on computed topography (CT) of the chest and/or pulmonary function testing (PFT). All patients were reviewed by RJ, BSB, VD and BB, and a total of 29 patients were selected after meeting all the inclusion and exclusion criteria.

### 2.3. Study Group Interventions

Patient characteristics were recorded and analyzed (Table 1). The 2017 EULAR/ACR classification of IIM was used for phenotypic determination [27]. The radiological patterns, distributions, and abnormalities were reviewed by BB and VD as described in the guidelines for diagnosis of idiopathic pulmonary fibrosis (IPF) by Raghu et al. [28]. Due to the retrospective nature of the study, a CT chest scan at the time of diagnosis of ILD was not available for all the patients. Therefore, the first available CT chest scan in the electronic medical records was taken as baseline. Fibrotic pattern on CT of the chest was defined by the presence of reticulation and at least one of the following: (1) septal thickening, (2) honeycombing, and (3) traction bronchiectasis. The pattern was otherwise defined as non-fibrotic. Treatment modalities utilized by the patient were recorded (Table 2). The clinical course based on the symptoms, supplemental oxygen requirement, ILD exacerbation rate, PFT and radiography of patients following treatment were recorded and analyzed. ILD exacerbation was defined as admissions to the hospital due to acute respiratory failure as determined by physicians in the clinical notes at the time of discharge. Treatment response was measured by similar criteria as established for IPF [29]. An improved response was defined by a decrease in the modified Medical Research Council (mMRC) dyspnea scale, reduction in the radiographic parenchymal abnormalities, physiologic improvement by >10% increase in the forced vital capacity (FVC) or >15% increase in the diffusion capacity of lungs for carbon monoxide (DLCO). A stable response was defined by no change in the mMRC dyspnea scale, 10% change in FVC, and <15% change in DLCO. On the contrary, failure to respond to treatment was defined as an increase in the mMRC dyspnea scale, an increase in the radiographic parenchymal abnormalities including progression to fibrotic pattern, and physiological deterioration by ≥10% decrease in the FVC or ≥15% decrease in DLCO. Cardiac involvement due to IIM was defined as outlined by Fairley et al. [30]. The following criteria were set for cardiac involvement in our study population: (i) troponin level ≥ 0.04 ng/mL, (ii) ejection fraction (EF) < 50%, (iii) diastolic dysfunction of any degree, and (iv) abnormal cardiac magnetic resonance imaging (MRI). While different types of troponins corelate variably with cardiac involvement in IIM, our institution and affiliates utilize troponin I with a cut-off of 0.03 ng/mL. Therefore, troponin I was used in this study to define cardiac involvement. Hypoalbuminemia was defined as an albumin level of less than 4 g/dL.

### 2.4. Outcomes

The primary outcome of this cross-sectional study was to assess the disease state and immunological and radiographic features of ILD with relation to underlying IIM in patients in the institution and its affiliated hospitals. The study determined the prevalence of the IIM phenotype and immune markers and radiographic patterns of ILD and sought to compare the results with other population studies. The secondary outcome of the study was to assess the disease progression of ILD in patients with IIM and the mortality rate and identify the risk factors for poor prognosis in this cohort.

### 2.5. Data Collection

All study records were securely stored in the hospital’s network of computers in the pulmonary office.

### 2.6. Statistical Methods

Descriptive analyses were performed for all outcome measures and endpoints. Continuous variables were reported as means with standard deviation or as medians with interquartile range. Categorical and binary data were reported in frequencies and percentages. For continuous variables, independent sample *t*-tests were used for normally distributed variables, while Mann–Whitney U tests were used for non-normally distributed variables. Categorical variables were analyzed using Pearson’s chi-square tests or Fisher’s exact tests where appropriate. All analysis was completed in the Statistical Package for Social Sciences (SPSS) for Windows version 28.0 (SPSS Inc., Chicago, IL, USA).

### 2.7. Bias

This cross-sectional study describes the rural Appalachian health status of IIM-ILD patients at a specified time-point. We attempted to minimize selection bias by having 3 independent reviewers determine the suitability of patients included in the study. Four reviewers performed independent chart reviews to extract and analyze data. Any disagreements were reconciled with a consensus decision. The retrospective nature of the study may lead to information bias. This study was performed to determine prevalence and association but not causality.

## 3. Results

### 3.1. Study Participants Characteristics

Baseline patient characteristics of the 29 patients included in the study are shown in Table 1. Most of the patients were White (89.70%, n = 26) and female (75.90%, n = 22) with a mean age of 60.17 ± 12.92 years and median body mass index (BMI) of 26.69 (23.45–39.29) kg/m^2^. The most common comorbidities were gastroesophageal reflux disease (34.48%, n = 10), followed by obstructive sleep apnea (24.13%, n = 7) and pulmonary hypertension (20.68%, n = 6). Eleven patients (37.93%) had a COVID-19 infection at some point in the disease course. Dyspnea (82.75%, n = 14) was the most common symptom at presentation. Other prevalent symptoms included a cough (37.93%, n = 11), fatigue (17.24%, n = 5), and joint pains (17.24%, n = 5). The duration of symptoms at the time of initial evaluation was 54.89 ± 79.96 months. The mean mMRC dyspnea scale was 2.00 ± 1.20. The median duration of follow-up pulmonary and rheumatology was 5.00 (1.25–8.00) and 6.00 (1.70–9.00) years, respectively. Data for oxygen requirement were available for 21 patients at the time of presentation; 6 (28.57%) required oxygen supplementation.

PFT for 20 patients were available prior to treatment initiation. Fifteen patients (75.00%) had a restrictive pattern on their PFT, one (5.00%) had an obstructive pattern, and four (20.00%) had normal PFT. The mean FVC and forced expiratory volume (FEV1) were 2.30 ± 0.70 L and 1.91 ± 0.51 L, respectively. The mean percent predicted DLCO was 54.73 ± 20.59% (n = 18).

### 3.2. Main Results

The most common phenotype of IIM was DM (51.72%, n = 15), followed by PM (27.58%, n = 8). Twenty patients had data available for autoantibody testing. The most common autoantibodies were the anti-Jo1 antibody (37.93%, n = 11) and the anti-Pl-7 antibody (6.89%, n = 2), whereas 17.24% patients (n = 5) had no autoantibody positive. The mean peak creatinine kinase (CK) was 1574.50 ± 1778.78 U/L and the mean aspartate and alanine aminotransferase levels were 93.96 ± 73.66 and 73.66 ± 68.02, respectively. Albumin levels were available for 28 patients. The median albumin level was 3.45 (3.00–3.78) g/dL, with 89.29% (n = 25) with hypoalbuminemia. Among 20 patients who had troponin levels, 50.00% (n = 10) had elevated levels with a median troponin level of 0.04 (0.02–0.14) ng/mL. A transthoracic echocardiogram was available for 24 patients. The median EF was 60.00 (55.50–65.00) percent with 3.04% (n = 1), and 20.83% (n = 5) patients had systolic and diastolic dysfunction. Only one patient had a cardiac MRI available which was normal. Fourteen patients (63.64%) underwent muscle biopsy for their diagnosis of IIM, whereas four patients (13.79%) underwent lung biopsy for a histopathologic evaluation of their ILD.

Treatment modalities used in the cohort are outlined in Table 2 and were available for 22 patients. Oral corticosteroids were utilized in 90.91% (n = 20) of patients during the disease course. Other anti-inflammatory medications included azathioprine (63.64%, n = 14), cyclophosphamide (59.10%, n = 13), and rituximab (36.36% n = 8). Anti-fibrotic therapy (Nintedanib) was implemented in 9.10% (n = 2).

**Figure 2:**
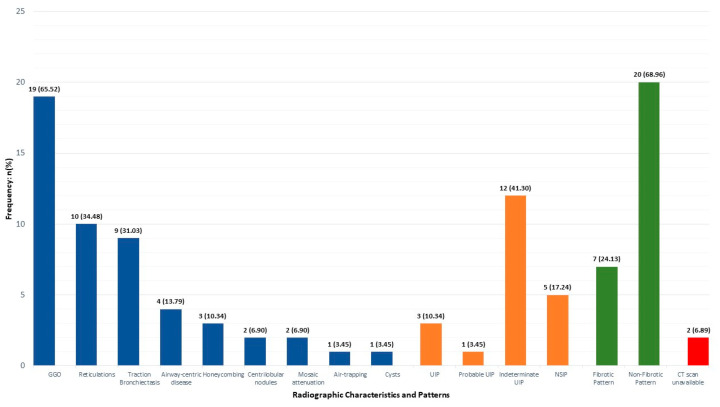
Computed tomography characteristics in study cohort. CT, computed tomography; NSIP, non-specific interstitial pneumonia; UIP, usual interstitial pneumonia.

The most common radiological findings were ground glass opacities (GGOs) (65.52%, n = 19), reticulations (34.48%, n = 10), and traction bronchiectasis (31.03%, n = 9). Indeterminate usual interstitial pneumonia (UIP) was the most common pattern (41.30%, n = 12), followed by non-specific interstitial pneumonia (NSIP) (17.24%, n = 5). Non-fibrotic changes were noted in 68.96% (n = 20), while 24.13% (n = 7) had fibrotic changes. The frequencies of other radiological findings and patterns are presented in Figure 2. CT chest scans of two patients were inaccessible.

The clinical course of the study patients is illustrated in Table 3. Symptoms progression, mMRC dyspnea scale, and PFT data were available for 18 patients, imaging data were available for 17 patients, and data for ILD exacerbation were available for 20 patients. Less than half of the patients had stable respiratory symptoms (n = 11). The reported mMRC remained stable over the course of the disease course in more than half of the patients (n = 15). Oxygen requirements remained stable in 41.18% (n = 12) patients. Radiographic changes remained stable in eight patients worsened in seven patients. PFT decline was noted in ten patients and four patients had either stable or improved PFT during the disease course.

Fourteen patients (66.66%) had ILD exacerbation during the study period. A comparison of patients with and without ILD exacerbation is listed in Table 4. Patients with PM were less likely to have ILD exacerbation (*p* = 0.025), while the prevalence of other IIM phenotypes and IIM autoantibodies was not statistically significant between the two groups. Patients with ILD exacerbation had a higher median peak C-reactive protein (CRP) value (*p* = 0.026) and lower median EF (*p* = 0.045). Radiographic characteristics and PFT findings did differ significantly between the two groups.

The overall mortality rate during the study period was 20.69% (n = 6). Table 5 lists a comparison between the patients who survived or died during the study. While the prevalence of hypoalbuminemia was not statistically different between the two groups, the mortality rate was higher in patients with lower median albumin levels (*p* < 0.001). Demographic and clinical features such as gender, age, smoking history, race, BMI, mMRC dyspnea scale, and duration of the symptoms did not differ between the two groups. IIM phenotypes and the prevalence of autoantibodies were also not significantly different between the two groups. Other laboratory testing such as peak CK, peak CRP, aldolase, troponin, and ESR levels were not statistically different between the two groups. The frequency of cardiac dysfunction was similar in both groups. Radiological features and patterns of ILD were not significantly different between the two groups.

## 4. Discussion

This study describes the clinical characteries, outcomes, and risk factors of poor prognosis in IIM-ILD patients in the rural Appalachian region of West Virginia. To our knowledge, this is the first study focusing on the rural population. Key findings of our study include female predominance and the relative infrequency of extrapulmonary symptoms: fatigue (17.24%), joint pain (17.24%), muscle weakness (10.34%), Raynaud’s phenomenon (10.34%), diffuse rash (6.89%) and heliotrope rash (3.44%). DM was the most common phenotype (51.72%), and anti-Jo1 was the most frequent autoantibody (55%) seen in this cohort. A predominant CT pattern was indeterminate for the UIP pattern (41.30%), and the worsening of PFTs was seen in 55.55% of patients in the study. ILD exacerbation and mortality were 60% and 20.69%, respectively, with significantly lower albumin levels observed in patients who died during the study period.

A higher proportion of female patients was seen in this study compared to European studies [31] but similar to the studies conducted in urban settings of the United States (US) [32]. Despite having one of the highest smoking rates in the region [33], smoking rates were relatively lower in our cohort. We found lower rates of extrapulmonary symptoms at the time of presentation in contrast to a study from urban regions of Japan which reported a relatively higher (45.6%) proportion of the patients presenting with arthralgia [34]. Similarly, another study showed that joint and muscular symptoms were present in 50% and 42% of the patients, respectively [35]. Patients with the anti-Jo1 antibody have been reported to have a higher proportion of musculoskeletal symptoms in multiple studies, as highlighted by Hallowell et al. [12]. Our study showed a low prevalence of musculoskeletal symptoms despite having a high proportion of patients with the anti-Jo1 antibody. Similar to our study, Raynaud’s phenomenon has been reported to be a less frequent symptom in patients affected with ILD in IIM [34,35]. A lower rate of extrapulmonary symptoms in our cohort may have contributed to a delayed presentation with a higher duration of symptoms at the first encounter. ILD was likely the presenting manifestation of an underlying immune disorder.

Both the IIM phenotype and associated autoantibodies have been reported as prognostic factors in IIM- ILD [36,37]. The most common IIM phenotype associated with ILD in our cohort was DM followed by PM. This finding is in keeping with an older study [38]. However, more recent studies report AS as one of the most common phenotypes in patients with IIM-ILD [22,39]. In contrast to our rural population, a study on a urban population in the U.S. reported polymyositis to be the most common phenotype [32]. Interestingly, the same studies [22,32,39] reported the anti-Jo1 antibody as the most common antibody in IIM-ILD, similar to our cohort. As the anti-Jo-1 antibody has been associated with the presence of ILD in IIM [40], it is expected to see high prevalence of its positivity in cohorts of patients exclusively with ILD [31]. A small cohort study of patients with ILD in IIM in Japan showed 71.42% patients to have the anti-Jo1 antibody [35].

Various radiological findings and patterns have been reported in IIM-ILD. The most common radiologic pattern found in our cohort was the indeterminate UIP. This contrasts the findings from prior studies which reported NSIP to be the most common pattern [17,22,31,41]. Similar to previous reports [42,43], ground glass opacities (GGOs) were the most common, while honeycombing and traction bronchiectasis were the least common CT findings in this cohort. Regardless of the overall CT pattern, isolated findings of GGOs have been associated with poor short-term outcomes in patients with myositis [44].

A majority of patients were started on the immunosuppression treatment in this study. Oral corticosteroids were the most commonly utilized modality of the treatment in addition to various other steroid-sparing immunosuppressive agents. No prospective trials have compared the efficacy of the various immunosuppressant agents for the treatment of ILD, and there are some data suggesting that most agents are interchangeable [12]. Despite immunosuppression, a significant proportion (55.55%) of patients in this study had worsening pulmonary function. In addition, only 10.34% noted improvement in their symptoms, 6.90% in their mMRC dyspnea scale, 3.45% in supplemental oxygen requirement and 6.90% in imaging. The low treatment response in our cohort is likely due to the high number of patients with the dermatomyositis phenotype. Previous studies have suggested that patients with dermatomyositis have a less favorable response rate to immunosuppression [11,32,45]. Antifibrotics were used in only two patients who met the criteria for fibrosis and had a progressive disease. While there are no studies available to specifically look at the efficacy of antifibrotics in IIM-associated ILD, they were shown to significantly lower the rate of pulmonary function decline in patients with progressive fibrosing ILD in the INBUILD trial which enrolled 170 patients with auto-immune disease-related ILD [46].

The ILD exacerbation rate was 66.66% in this cohort which is significantly higher than that which was previously reported in a large case-control study [47]. It is unclear why there was such a high rate of ILD exacerbation in our study, but the reason may stem from the lack of definitive criteria for ILD exacerbation in IIM, the confounding presentation with other diseases such heart failure, differences in physician practices, and the lack of referral to tertiary care centers with ILD experts. Liang et al. [47] reported that patients who were admitted with ILD exacerbation tend to have higher CRP levels, lower DLCO% on PFT, and higher prevalence of the ADM phenotype. Similar to their study, peak CRP levels were also higher in patients with ILD exacerbation in our cohort. On the contrary, the rate of ILD exacerbation did not differ by PFT findings. Interestingly, we found lower exacerbation rates in patients with the PM phenotype.

The mortality rate in our cohort of patients with ILD in IIM was 20.69% which was higher than what was observed in a study conducted at a large urban tertiary care center in the U.S. [13]. Similarly, it was also higher than that from other similar- and larger-sized cohort studies from Europe [22,48]. A study on the Chinese population reported hypoalbuminemia and cardiac dysfunction as poor prognostic factors [37]. In this study, hypoalbuminemia was very prevalent (89.29%). While the prevalence of hypoalbuminemia was not statistically different between patients who survived and those who did not, albumin levels were significantly lower in the patients who died during the study period. Albumin level is a well-established marker of nutritional status [49]. Poor nutritional status of this cohort may have contributed to the observed increased mortality. Cardiac disease was not prevalent in our cohort; therefore, it is difficult to conclude the significance of cardiac involvement with regard to poor outcomes.

Multiple poor prognostic factors such as older age, amyopathic phenotype, anti-MDA5 antibody, and lower CK levels have been reported previously [11,31,38,50]. This study did not find any significant association of mortality with the myositis phenotype, which is similar to the study by Johnson et al. [13]. Only one patient in the study had the amyopathic subtype; therefore, an association with mortality could not be established. This study also did not show any association of mortality with age, the duration and severity of symptoms at presentation, and the antibody type. Overall, this study is limited by its small sample size which may explain the lack of statistical significance.

The characteristics and outcomes of patients with IIM-associated ILD in the rural Appalachian region are dissimilar from previously published cohort studies of general and urban populations, posing significant diagnostic, management, and treatment challenges. Given the low prevalence of extrapulmonary symptoms, it is important to note that ILD can be the first manifestation of the disease. A good understanding of clinical, immunological, and radiologic features of IIM-ILD are crucial to improve outcomes. Given the complexity involved in the diagnosis of IIM-associated ILD and its exacerbation, early referral to tertiary care ILD centers with multidisciplinary team support can provide an early diagnosis and appropriate treatment for these patients. A suboptimal response to immunosuppression was observed in this cohort similar to other studies which signifies the need for larger studies to evaluate the role of immunosuppression and antifibrotics in IIM-ILD. Lastly, poor nutritional status in our cohort, as denoted by albumin levels, was associated with increased mortality in our cohort. Early referral to a dietary specialist for nutritional care for these patients may be key to improving outcomes.

Limitations: this study is limited in its generalizability and ability to generate statistically significant results due to the small sample size. However, the rarity of IIM-ILD and the shifting diagnostic criteria for IIM likely limited recruitment. The retrospective nature of the study may potentially lead to selection bias. Larger prospective studies are needed to determine prognostic markers that may alter the course and outcomes of patients with IIM-ILD.

## 5. Conclusions

The most common IIM phenotype associated with ILD was dermatomyositis, whereas the most common autoantibody was the anti-Jo1 antibody. Fewer patients had extrapulmonary manifestation in our cohort, which likely influenced delayed presentation. The most common radiological pattern and findings were indeterminate UIP and GGO, respectively. The ILD exacerbation and mortality rate was higher in our cohort as compared to previous studies. The albumin level, a marker of nutritional status, was significantly lower in the patients who died during the study period.

## Figures and Tables

**Table 1:** Baseline patient characteristics.

Variables	n = 29
Age: mean (SD)	60.17 (12.92)
Female: n (%)	22 (75.90)
Never-smoker: n (%)	19 (65.50)
Former smoker: n (%)	10 (34.50)
Amount in pack years ^a^: mean (SD)	24.89 (17.47)
Race:	
White: n (%)	26 (89.70)
African American: n (%)	3 (10.30)
BMI median (IQR)	26.39 (23.45, 39.29)
History of COVID-19 infection: n (%)	11 (37.93)
Dyspnea: n (%)	24 (82.75)
Cough: n (%)	11 (37.93)
Fatigue, joint pain: n (%)	5 (17.24)
Muscle weakness, Raynaud’s phenomenon: n (%)	3 (10.34)
Dysphagia: n (%)	2 (6.89)
Diffuse rash: n (%)	2 (6.89)
Heliotrope rash: n (%)	1 (3.44)
Night sweats, decreased appetite, Gottron’s papules: n (%)	0 (0)
mMRC dyspnea scale on presentation: mean (SD)	2.00 (1.20)
Duration of symptoms at first encounter in months: mean (SD)	54.89 (79.96)
Oxygen dependence at rest/exercise ^e^: n (%)	6 (28.57)
Idiopathic inflammatory myopathy phenotype:	
Dermatomyositis: n (%)	15 (51.72)
Polymyositis: n (%)	8 (27.58)
Anti-synthetase syndrome: n (%)	5 (17.24)
Amyopathic dermatomyositis: n (%)	1 (3.44)
Gastroesophageal reflux disease: n (%)	10 (34.48)
Obstructive sleep apnea: n (%)	7 (24.13)
Pulmonary hypertension: n (%)	6 (20.68)
Asthma: n (%)	5 (17.24)
Other connective tissue disease: n (%)	4 (13.79)
Chronic obstructive pulmonary disease: n (%)	3 (10.34)
Congestive heart failure, any cancer, DVT/PE: n (%)	2 (6.89)
Autoantibodies ^f^: n (%)	
Anti-Jo1 Ab	11 (55.00)
None	5 (25.00)
PI-7 Ab	2 (10.00)
NXP2 Ab, anti-SS-A 52 kD Ab, anti-PM/Scl Ab	1 (5.00)
Chronic kidney disease, cirrhosis: n (%)	1 (3.44)
Peak Creatinine Kinase ^b^: mean (SD)	1574.50 (1778.78)
Aspartate aminotransferase ^i^: median (IQR)	58.00 (30.00, 121.00)
Alanine aminotransferase ^i:^ median (IQR)	46.00 (31.00, 108.00)
Peak C-reactive protein ^c^: mean (SD)	81.76 (119.48)
Aldolase ^d^: median (IQR)	14.15 (8.10, 37.40)
Erythrocyte sedimentation rate: mean (SD) ^d^	32.21 (33.13)
Albumin ^b^: median (IQR)	3.45 (3.00, 3.78)
Hypoalbuminemia ^b^: n (%)	25 (89.29)
Troponin (ng/mL) ^f^: median (IQR)	0.04 (0.02, 0.14)
Troponemia ^f^: n (%)	10 (50.00)
Ejection Fraction ^d^: median (IQR)	60.00 (55.50, 65.00)
Systolic heart dysfunction (EF < 50) ^d^: n (%)	1 (3.40)
Diastolic heart dysfunction ^d^: n (%)	5 (20.83)
Forced vital capacity ^f^, liters: mean (SD)	2.30 (0.70)
Forced vital capacity, percent predicted ^f^: mean (SD)	62.85 (14.58)
Forced expiratory volume in 1 s ^f^, Liters: mean (SD)	1.91 (0.51)
Forced expiratory volume in 1 s, percent predicted ^f^: mean (SD) ^f^	67.22 (14.88)
FEV1/FVC ^e^: mean (SD)	82.29 (5.96)
TLC ^g^: mean (SD)	4.29 (1.18)
TLC ^h^ % predicted: mean (SD)	73.63 (16.62)
DLCO, mL/min/mmHg: mean (SD) ^g^	16.51 (6.50)
DLCO, percent predicted: mean (SD) ^g^	54.73 (20.59)
Restrictive pattern on pulmonary function testing: n (%) ^f^	15 (75.00)
Obstructive pattern on pulmonary function testing: n (%) ^f^	1 (5.00)
Normal pattern on pulmonary function testing: n (%) ^f^	4 (20.00)
Lung biopsy: n (%)	4 (13.79)

^a^ n = 9; ^b^ n = 28; ^c^ n = 25; ^d^ n = 24; ^e^ n = 21; ^f^ n = 20; ^g^ n = 18; ^h^ n = 17; ^i^ n = 27. BMI, body mass index; DLCO, diffusion capacity for carbon monoxide; DVT, deep venous thrombosis; FEV1/FVC, ratio of forced expiratory volume in 1 s and forced vital capacity; mMRC, modified Medical Research Council dyspnea scale; PE, pulmonary embolism; TLC, total lung capacity.

**Table 2:** Treatment modalities for study population *.

Medication n (%)	n = 22
Prednisone	20 (90.91)
Azathioprine	14 (63.64)
Cyclophosphamide	13 (59.09)
Rituximab	8 (36.36)
Methotrexate	4 (18.18)
Nintedanib	2 (9.09)

* Medications group not mutually exclusive.

**Table 3:** Clinical course of study population.

Clinical Parameter	n = 29
Symptoms progression: n (%)	
Improved	3 (10.34)
Worsened	6 (20.69)
Stable	11 (37.93)
Follow-up symptoms not available	9 (31.03)
mMRC dyspnea scale: n (%)	
Improved	2 (6.90)
Worsened	2 (6.90)
Stable	15 (51.72)
Follow-up mMRC not available	10 (34.48)
Oxygen requirement: n (%)	
Improved	1 (3.45)
Worsened	7 (24.14)
Stable	12 (41.38)
Follow-up oxygen requirement not available	9 (31.03)
Imaging: n (%)	
Improved	2 (6.90)
Worsened	7 (24.14)
Stable	8 (27.59)
Follow-up imaging not available	12 (41.38)
PFT: n (%)	
Improved	4 (22.22)
Worsened	10 (55.55)
Stable	4 (22.22)
Follow-up PFT not available	11 (37.93)
ILD Exacerbation ^a^: n (%)	14 (66.66)
Mortality: n (%)	6 (20.69)

^a^ n = 21. mMRC, modified Medical Research Council dyspnea scale; ILD, interstitial lung disease; PFT, pulmonary function test.

**Table 4:** Comparison of patients with and without ILD exacerbation.

Baseline Variables	ILD Exacerbation	No ILD Exacerbation	*p*-Value
	n = 14	n = 7	
Age: mean (SD)	58.64 (15.53)	61.00 (8.64)	0.715
Female: n (%)	12 (85.71)	4 (57.14)	0.280
Former smoker: n (%)	2 (14.29)	2 (28.57)	0.547
Race: White: n (%)	13 (92.86)	6 (85.71)	1.000
BMI: median (IQR)	25.05 (22.44, 42.67)	26.54 (25.07, 33.95)	0.913
IIM phenotype:			
Dermatomyositis: n (%)	8 (57.14)	3 (42.86)	0.659
Polymyositis: n (%)	1 (7.14)	4 (57.14)	0.025
Anti-synthetase syndrome: n (%)	5 (35.71)	0	0.123
Amyopathic myositis: n (%)	0	0	none
Autoantibodies: n (%)			
None	2 (16.67) ^e^	2 (66.67) ^i^	0.154
Jo1 Ab	7 (58.33) ^e^	0 ^i^	0.200
NXP2 Ab	1 (8.33) ^e^	0 ^i^	1.000
PI-7 Ab	2 (16.67) ^e^	0 ^i^	1.000
Anti-SS-A 52 kD Ab	0 ^e^	1 (33.33) ^i^	0.200
Anti-PM/Scl Ab	0 ^e^	1 (33.33) ^i^	0.200
History of COVID-19: n (%)	6 (42.86)	3 (42.86)	1.000
Family history of ILD: n (%)	0	0	none
CHF: n (%)	0	0	none
GERD: n (%)	6 (46.15) ^c^	2 (28.57)	0.642
PH: n (%)	4 (30.77) ^c^	0	0.155
mMRC dyspnea scale on presentation: mean (SD)	2.07 (1.33)	2.43 (1.13)	0.551
Duration of symptoms at first encounter in months: median (IQR)	12.00 (3.00, 90.00)	51.00 (5.00, 201.00) ^a^	0.239
Peak CK: median (IQR)	502.00 (108.50, 1625.25)	3744.00 (363.00, 5066.00)	0.110
CRP peak: median (IQR)	56.40 (20.00, 139.35) ^c^	6.10 (2.85, 45.05) ^b^	0.026
Aldolase: median (IQR)	15.30 (7.25, 34.10) ^c^	18.20 (11.40, 50.25) ^a^	0.521
ESR: median (IQR)	22.00 (9.00, 43.00) ^f^	13.00 (5.25, 19.50) ^a^	0.149
Albumin: median (IQR)	3.60 (3.15, 3.75)	3.85 (3.38, 3.93) ^a^	0.312
Hypoalbuminemia: n (%)	12 (85.71)	5 (83.33) ^a^	1.000
Troponin (ng/mL): median (IQR)	0.03 (0.02, 0.08) ^e^	0.15 ^d^	none
Troponin ≥ 0.04: n (%)	5 (41.67) ^e^	1 ^d^	none
EF: median (IQR)	60.00 (58.00, 64.50)	65.00 (64.25, 71.75) ^j^	0.045
Systolic heart dysfunction (EF < 50%): n (%)	0 ^c^	0 ^j^	none
Diastolic dysfunction: n (%)	4 (30.77) ^c^	0 ^j^	0.323
DLCO, mL/min/mmHg: mean (SD)	14.72 (5.91) ^g^	20.86 (3.33) ^b^	0.313
DLCO, percent predicted: mean (SD)	52.11 (15.58) ^h^	66.24 (24.88) ^b^	0.496
O2 requirement at rest: n (%)	3 (21.43)	1 (14.29)	1.000
O2 requirement during exercise: n (%)	4 (28.57)	2 (28.57)	1.000
PFT: n (%)			
Improved	2 (18.18) ^f^	0 ^j^	1.000
Worsened	7 (63.64) ^f^	3 (75.00) ^j^	1.000
Stable	2 (18.18) ^f^	1 (25.00) ^j^	1.000
Mortality: n (%)	2 (14.29)	0	0.189
CT Chest Findings			
UIP: n (%)	1 (7.14)	1 (14.29)	1.000
Probable UIP: n (%)	2 (14.29)	0	0.533
Indeterminate UIP: n (%)	7 (50.00)	4 (57.14)	1.000
NSIP: n (%)	4 (28.57)	0	0.255

^a^ n = 6; ^b^ n = 5; ^c^ n = 13; ^d^ n = 1; ^e^ n = 12; ^f^ n = 11; ^g^ n = 7; ^h^ n = 9; ^i^ n = 3; ^j^ n = 4; none = unable to run comparison due to insufficient sample size. AST; aspartate aminotransferase, ALT; alanine aminotransferase; BMI, body mass index; CK, creatinine kinase; CRP, C-reactive protein; CT, computed tomography; DLCO, diffusion capacity of lung for carbon monoxide; ESR, erythrocyte sedimentation rate; ILD, interstitial lung disease; IQR, inter-quartile range; mMRC, modified Medical Research Council dyspnea scale; NSIP, non-specific interstitial pneumonia; UIP, usual interstitial pneumonia.

**Table 5:** Comparison of patients who died or survived.

Baseline Variables	Survivors	Non-Survivors	*p*-Value
	n = 23	n = 6	
Age: median (IQR)	57.00 (49.00, 66.00)	68.00 (57.25, 72.50)	0.232
Female: n (%)	18 (78.26)	4 (66.67)	0.612
Former smoker: n (%)	7 (30.43)	3 (50.00)	0.633
Race: White: n (%)	20 (86.96)	3 (50.00)	1.000
BMI median (IQR)	26.54 (23.99, 39.92)	24.93 (21.62, 34.43)	0.477
IIM phenotype:			
Dermatomyositis: n (%)	12 (52.17)	3 (50.00)	1.000
Polymyositis: n (%)	7 (30.43)	1 (16.67)	0.647
Anti-synthetase syndrome: n (%)	4 (17.39)	1 (16.67)	1.000
Amyopathic dermatomyositis: n (%)	0	1 (16.67)	0.207
Autoantibodies: n (%)			
None	5 (17.3)		
Jo1 Ab	8 (53.33) ^i^	3 (60.00) ^j^	1.000
NXP2 Ab	1 (6.67) ^i^	0 ^j^	1.000
PI-7 Ab	1 (6.67) ^i^	1 (20.00) ^j^	0.447
Anti-SS-A 52 kD Ab	1 (6.67) ^i^	0 ^j^	1.000
Anti-PM/Scl Ab	1 (6.67) ^i^	0 ^j^	1.000
History of COVID-19: n (%)	11 (47.83)	0	0.058
Family history of ILD: n (%)	0	0	none
CHF: n (%)	1 (4.55) ^b^	1 (16.67)	0.389
GERD: n (%)	8 (36.36) ^b^	2 (33.33)	1.000
PH: n (%)	3 (13.04)	3 (50.00)	0.091
mMRC dyspnea scale on presentation: median (IQR)	2.00 (1.00, 3.00)	2.50 (1.00, 2.50)	0.511
Duration of symptoms at first encounter in months: median (IQR)	24.00 (4.50, 96.00) ^a^	12.00 (4.50, 47.25)	0.629
Peak CK: median (IQR)	1022.50 (300.00, 2232.50) ^b^	883.50 (102.75, 2368.00)	0.723
CRP peak: median (IQR)	50.60 (11.10, 79.00) ^c^	97.00 (9.53, 270.00)	0.246
Aldolase: median (IQR)	12.85 (7.80, 37.40) ^d^	27.60 (11.35, 37.78) ^e^	0.525
ESR: median (IQR)	18.00 (9.00, 38.00) ^c^	33.00 (19.50, 40.00) ^f^	0.446
Albumin: median (IQR)	3.60 (3.18, 3.90) ^b^	2.20 (1.73, 3.03)	<0.001
Hypoalbuminemia: n (%)	19 (82.60) ^b^	6 (100)	1.000
Troponin (ng/mL): median (IQR)	0.03 (0.02, 0.08) ^g^	0.07 (0.04, 0.19)	0.179
Troponin ≥ 0.04: n (%)	5 (35.71)	5 (83.33)	0.141
EF: median (IQR)	60.00 (58.50, 65.00) ^k^	56.50 (46.25, 65.00)	0.137
Systolic heart dysfunction (EF < 50%): n (%)	0 ^k^	1 (16.67)	0.250
Diastolic dysfunction: n (%)	4 (22.22) ^k^	1 (16.67)	1.000
O2 requirement at rest: n (%)	3 (15.79) ^c^	1 (50.00) ^h^	0.352
O2 requirement during exercise: n (%)	4 (21.05) ^c^	2 (100.00) ^h^	0.071
ILD exacerbation: n (%)	11 (57.89) ^c^	2 (66.6) ^m^	0.50
CT Chest Findings			
UIP: n (%)	2 (9.50)	1 (16.6) ^l^	0.54
Probable UIP: n (%)	1 (4.76)	0 (0)	1.00
Indeterminate UIP: n (%)	9 (42.85)	3 (50.00)	1.00
NSIP: n (%)	3 (14.28)	2 (33.33)	0.30

^a^ n = 21; ^b^ n = 22; ^c^ n = 19; ^d^ n = 20; ^e^ n = 4; ^f^ n = 5; ^g^ n = 14; ^h^ n = 2; ^i^ n = 15; ^j^ n = 5; ^k^ n = 18; ^l^ n = 6; ^m^ n = 3; none = unable to run comparison due to insufficient sample size. AST, aspartate aminotransferase; ALT, alanine aminotransferase; BMI, body mass index; CK, creatinine kinase; CRP, C-reactive protein; CT, computed tomography; DLCO, diffusion capacity of lung for carbon monoxide; ESR, erythrocyte sedimentation rate; ILD, interstitial lung disease; IQR, inter-quartile range; mMRC, modified Medical Research Council dyspnea scale; NSIP, non-specific interstitial pneumonia; UIP, usual interstitial pneumonia.

## Data Availability

All data and analysis generated during the study are available from the corresponding author upon request. The data are not publicly available due to containing information that could compromise the patients’ privacy.

## Abbreviations

ACR	American College of Rheumatology
ADM	Amyopathic dermatomyositis
AS	Anti-synthetase syndrome
BMI	Body mass index
CK	Creatinine kinase
CRP	C-reactive protein
CT	Computed topography
DLCO	Diffusion capacity of lung for carbon monoxide
DM	Dermatomyositis
EF	Ejection fraction
ESR	Erythrocyte sedimentation rate
EULAR	European league against rheumatism
FEV1	Forced expiratory volume in first second
FVC	Forced vital capacity
GGO	Ground glass opacity
IIM	Idiopathic inflammatory myopathy
ILD	Interstitial lung disease
IPF	Idiopathic pulmonary fibrosis
IRB	Institutional Review Board
mMRC	modified Medical Research Council
MRI	Magnetic resonance imaging
NSIP	Non-specific interstitial pneumonia
PFT	Pulmonary function test
PM	Polymyositis
SPSS	Statistical Package for Social Sciences
UIP	Usual interstitial pneumonia
US	United States
WVU	West Virginia University
